# Sex-specific alterations in glucose homeostasis and metabolic parameters during ageing of caspase-2-deficient mice

**DOI:** 10.1038/cddiscovery.2016.9

**Published:** 2016-02-29

**Authors:** C H Wilson, A Nikolic, S J Kentish, S Shalini, G Hatzinikolas, A J Page, L Dorstyn, S Kumar

**Affiliations:** 1 Centre for Cancer Biology, University of South Australia, Adelaide, SA, Australia; 2 Discipline of Medicine, University of Adelaide, Adelaide, SA, Australia; 3 South Australian Health and Medical Research Institute, Adelaide, SA, Australia

## Abstract

Gender-specific differences are commonly found in metabolic pathways and in response to nutritional manipulation. Previously, we identified a role for caspase-2 in age-related glucose homeostasis and lipid metabolism using male *caspase-2*-deficient (*Casp2*^*−/−*^) mice. Here we show that the resistance to age-induced glucose tolerance does not occur in female *Casp2*^*−/−*^ mice and it appears to be independent of insulin sensitivity in males. Using fasting (18 h) as a means to further investigate the role of caspase-2 in energy and lipid metabolism, we identified sex-specific differences in the fasting response and lipid mobilization. In aged (18–22 months) male *Casp2*^*−/−*^ mice, a significant decrease in fasting liver mass, but not total body weight, was observed while in females, total body weight, but not liver mass, was reduced when compared with wild-type (WT) animals. Fasting-induced lipolysis of adipose tissue was enhanced in male *Casp2*^*−/−*^ mice as indicated by a significant reduction in white adipocyte cell size, and increased serum-free fatty acids. In females, white adipocyte cell size was significantly smaller in both fed and fasted *Casp2*^*−/−*^ mice. No difference in fasting-induced hepatosteatosis was observed in the absence of caspase-2. Further analysis of white adipose tissue (WAT) indicated that female *Casp2*^*−/−*^ mice may have enhanced fatty acid recycling and metabolism with expression of genes involved in glyceroneogenesis and fatty acid oxidation increased. Loss of *Casp2* also increased fasting-induced autophagy in both male and female liver and in female skeletal muscle. Our observations suggest that caspase-2 can regulate glucose homeostasis and lipid metabolism in a tissue and sex-specific manner.

## Introduction

Metabolic processes including lipid metabolism and their contribution to body composition are known to be regulated in a gender-specific manner.^1^ Caspase-2 is a proteolytic cell-death enzyme known to have both apoptotic and non-apoptotic roles affecting the stress response, genomic stability, tumorigenesis and ageing.^2–7^ Although a function of caspase-2 in metabolism has been suggested previously,^8,9^ information on sex-specific differences in *caspase-2*-deficient (*Casp2*^*−/−*^) mice have not been well documented. In the absence of exogenous stressors, *Casp2*^*−/−*^ mice display only a mild phenotype of enhanced premature ageing and altered body composition including reduced maximal body weight, fat, bone mass and a decrease in epidermal skeletal muscle mass (males only).^8,9^ Previously, we have shown that the ageing phenotype is due, in part, to an increase in hepatic oxidative stress-induced damage and impaired antioxidant response,^7,9^ but the reason for altered body composition is yet to be determined.

Recently, we identified a role for caspase-2 in the regulation of age-related proteostasis, energy metabolism, lipid metabolism and glucose homeostasis.^10^ We observed altered liver mitochondrial function in young (6- to 9-week-old mice) and aged (18–24 month) *Casp2*^*−/−*^ mice.^10^ In addition, we found a reduction in blood glucose levels in the fed and fasted state and improved glucose tolerance in aged male *Casp2*^*−/−*^ mice.^10^

Caspase-2 has been linked to lipid metabolism by a number of other studies, including evidence of transcriptional regulation of human *CASP2* sterol regulatory element-binding protein 2,^11^ altered caspase-2 expression following high-fat diet feeding^12^ and protection from type-I diabetes-induced bone marrow adiposity in *Casp2*^*−/−*^ mice.^13^ In addition, activation of caspase-2 may be important for lipoapoptosis (excess lipid-induced cell death) as identified in studies using *Xenopus* oocytes and human HepG2 cells.^14^ Despite these findings, no study has as yet specifically focused on determining the *in vivo* role of caspase-2 in lipid metabolism.

Fasting is a form of nutritional and metabolic stress and used as a means to study lipid metabolism. During fasting, nutritional deprivation first results in depletion of hepatic glycogen stores followed by lipolysis of white adipose tissue (WAT) and eventual breakdown of muscle protein stores to ensure that energy demands are continually met for survival.^15–17^ WAT lipolysis results in the release of free fatty acids (FFAs) and glycerol into circulation for uptake and utilization by the liver and skeletal muscle. Liver and skeletal muscle can directly oxidize FFAs for energy supply.^15,16,18^ The liver also metabolizes FFAs to ketone bodies for release and use by other organs, while glycerol is primarily used for hepatic glucose production via gluconeogenesis.^16,17,19^

Autophagy is also an important cell survival process, which is induced following starvation to promote recycling of intracellular components (including proteins and lipids) to provide substrates for energy production.^20^ Fasting-induced autophagic proteolysis of skeletal muscle and liver is important for release of amino acids into the circulation and has been shown to be important in the maintenance of blood glucose levels.^21,22^ Interestingly, there is evidence demonstrating that steady-state basal levels of autophagy are enhanced in the absence of *Casp2*.^23,24^

In this study, we aimed to investigate the role of caspase-2 in glucose and energy homeostasis, lipid metabolism and fuel utilization through nutritional deprivation studies. In addition, we have performed the first study to specially look at sex-specific differences in the metabolism of aged *Casp2*^*−/−*^ mice and investigated *in vivo* autophagic flux in liver and skeletal muscle tissue.

## Results

### Caspase-2 alters glucose tolerance of aged mice in a sex-dependent manner

**Figure 1:**
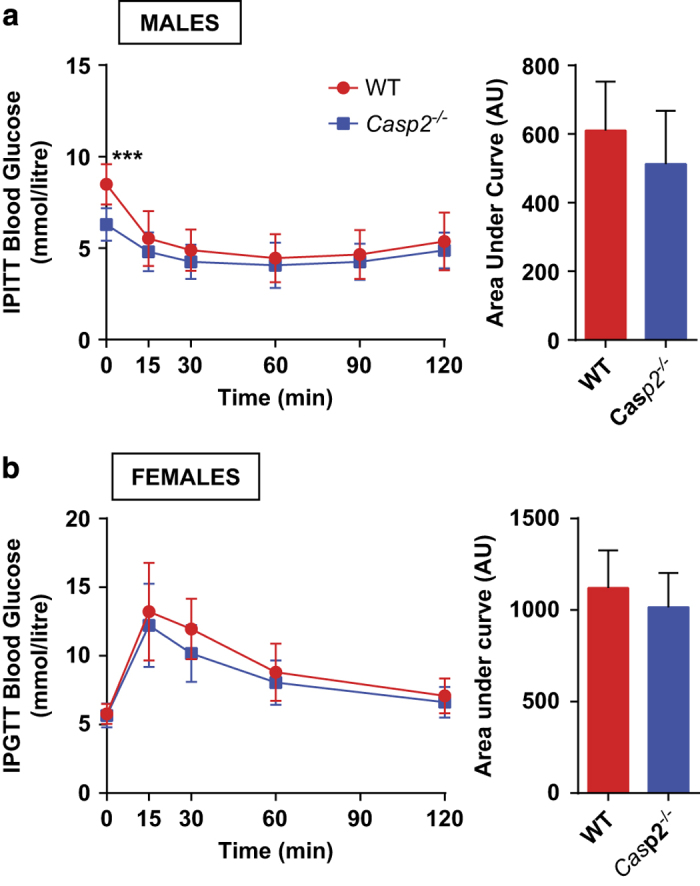
Improved glucose tolerance in aged *Casp2*^*−/−*^ mice does not occur in females and is insulin-independent in males. (**a**) Insulin tolerance test in aged male and (**b**) aged female WT and *Casp2*^*−/−*^ mice. Values are mean±S.D. (*n*=8–10). Unpaired *t*-test: ***P<0.01.

Previously, we observed reduced fed and fasted blood glucose levels in aged male *Casp2*^*−/−*^ mice and protection from age-induced glucose intolerance that was observed in WT mice following glucose tolerance testing.^10^ Although aged *Casp2*^*−/−*^ mice have increased oxidative damage, studies have shown that increased ROS in mice can result in enhanced insulin sensitivity.^18^ To determine if this was a reason for improved glucose tolerance in aged male *Casp2*^*−/−*^ mice, insulin tolerance testing was performed following a 6-h fast. Although blood glucose was significantly lower in male *Casp2*^*−/−*^ mice (6.29±0.89 mmol/l *versus* 8.49±1.1 mmol/l in WT mice) following fasting, there was no difference in the response to intraperitoneal (i.p.) injection of insulin (Figure 1a). As our previous findings were in male mice only, we performed glucose tolerance testing on aged female *Casp2*^*−/−*^ mice but found no difference compared to WT mice (Figure 1b). These results suggest that caspase-2 influences glucose homeostasis in a sex-specific manner independent of insulin signaling.

### Sex-specific alterations in fasting-induced lipolysis of adipose tissue in *Casp2*^*−/−*^ mice

**Figure 2:**
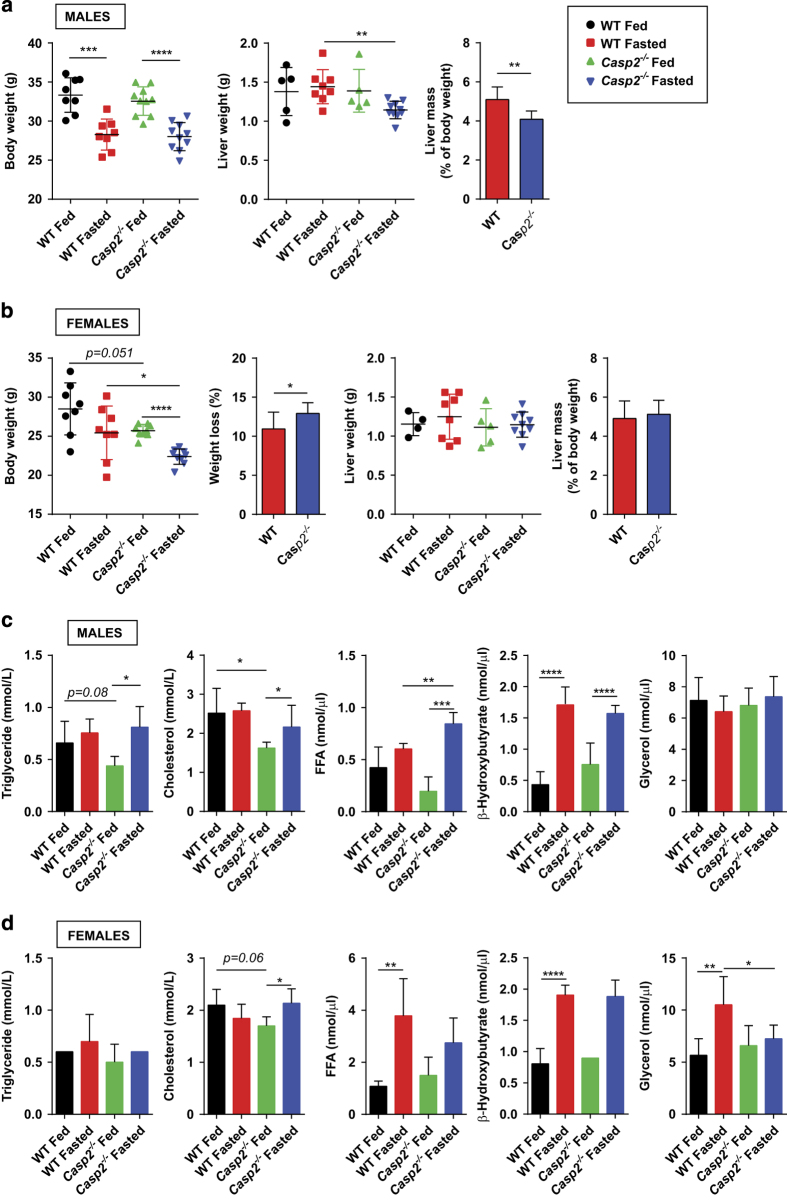
Fasting induces sex-specific differences in body weight loss and liver mass of aged *Casp2*^*−/−*^ mice. Total body weight and liver mass of (**a**) male and (**b**) female fed and fasted WT and *Casp2*^*−/−*^ mice. Bar graph display percentage of body weight loss following fasting. Serum parameters of (**c**) male and (**d**) female fed and fasted mice. Values are mean±S.D. (*n*=4–9). Unpaired *t-*test: *P<0.05, **P<0.01, ***P<0.001 and ****P<0.0001.

Glucose homeostasis can be related to body composition. As a leaner phenotype has been previously observed in aged *Casp2*^*−/−*^ mice,^8,9^ we wanted to investigate this further and focus on the role of caspase-2 in lipid metabolism. First, the possible contribution of food intake to body composition was determined but no difference in average daily food intake was observed (Supplementary Figure S1). To determine the contribution of altered lipid mobilization and utilization to body composition in *Casp2*^*−/−*^ mice, animals were fasted for 18 h with *ad libitum* access to water. Fasting body weights were significantly reduced in all mice except WT females (Figures 2a and b). In males, *Casp2* deficiency did not alter the total amount of body weight lost. In females, *Casp2*^*−/−*^ mice had a trend toward lower body weight in both the fed and fasted state. The percentage of body weight lost by female *Casp2*^*−/−*^ mice following fasting was significantly higher than WT mice (Figure 2b). Interestingly, fasting liver weight was significantly reduced in male but not in female *Casp2*^*−/−*^ mice (Figure 2b). These findings suggested that caspase-2 influences the response to fasting in a sex-specific manner.

In the fed state, serum cholesterol levels were significantly reduced in *Casp2*^*−/−*^ mice, independent of sex and increased upon fasting (Figures 2c and d). This increase was not observed in WT mice. Levels of serum triglycerides followed a similar pattern in male *Casp2*^*−/−*^ mice (Figure 2e). Serum FFAs and *β*-hydroxybutyrate (*β*-HB), a ketone body produced in liver from *β*-oxidation of fatty acids, were increased by fasting in all groups. However, the increase in fasted serum FFA was significantly higher in male *Casp2*^*−/−*^ compared to WT mice (Figures 2c and d). Fasting significantly increased serum glycerol levels in female WT mice only (Figure 2d). This suggests that caspase-2 influences cholesterol metabolism independent of sex.

**Figure 3:**
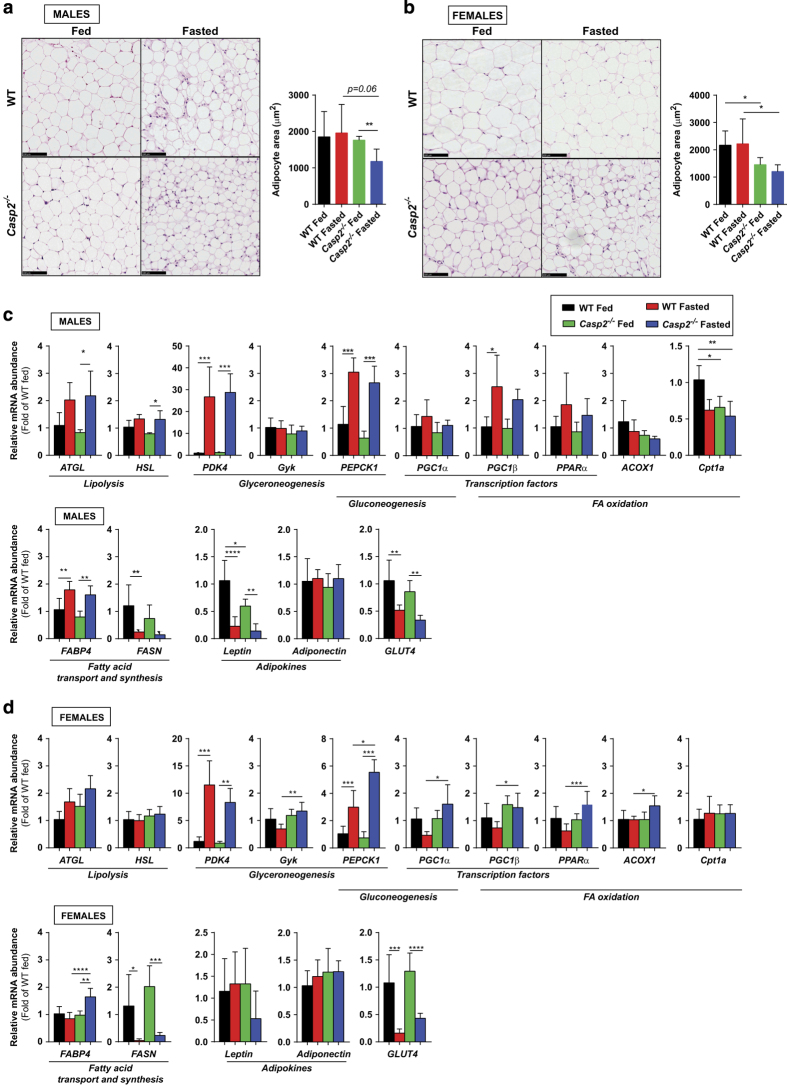
Lipolysis of adipose tissue is increased in male *Casp2*^*−/−*^ mice. Histological analyses of gWAT from (**a**) male and (**b**) female fed and fasted WT and *Casp2*^*−/−*^ mice (×20 magnification). Bar graphs show mean adipocyte size per field of view as measured in ImageJ from >200 cells per mouse from three random fields of view (*n*=4–8). Metabolic gene expression in gWAT of (**c**) male and (**d**) female fed and fasted mice. Values are mean±S.D. (*n*=4–8). One-way ANOVA (**c** and **d**) or unpaired *t*-test (**a** and **b**): *P<0.05, **P<0.01, ***P<0.001 and ****P<0.0001. Scale bar, 100 *μ*m.

To meet energy demands, prolonged fasting induces lipolysis of WAT to release glycerol and FFAs into circulation for uptake by the liver and oxidative tissues for fuel utilization. Histological analysis of gonadal WAT (gWAT) revealed that 18 h of fasting induced a significant decrease in white adipocyte cell size in male but not in female *Casp2*^*−/−*^ mice (Figures 3a and b). In female *Casp2*^*−/−*^ mice, adipocyte size of gWAT was significantly smaller compared to WT mice in both the fed and fasted state (Figure 3b). Histological analysis of interscapular brown adipose tissue (iBAT) revealed a decrease in lipid-filled vacuoles in fasted male *Casp2*^*−/−*^ mice but not female or WT mice (Supplementary Figure S2). Combined with the higher level of circulating FFAs, the decrease in adipocyte size in aged *Casp2*^*−/−*^ male mice is indicative of increased lipolysis of adipose tissue.

### Caspase-2-deficiency alters metabolic gene expression in fed and fasted WAT

To further examine this altered lipolysis in *Casp2*^*−/−*^ mice, we assessed gene expression of two of the main lipolytic enzymes, adipose triglyceride lipase (ATGL) and hormone-sensitive lipase (HSL), and found significantly increased levels in fasted male *Casp2*^*−/−*^ mice (Figure 3c). In addition, pyruvate dehydrogenase kinase 4 (PDK4), a key enzyme of glyceroneogenesis induced by fasting,^25^ was significantly increased in fasted mice as was the dual gluconeogenic and glyceroneogenic enzyme, phosphoenolpyruvate carboxykinase (PEPCK1; Figures 3c and d). In females, fasting PEPCK1 levels were significantly higher in *Casp2*^*−/−*^ mice than WT mice and were accompanied by significant increase in glycerol kinase (GYK) expression (Figure 3d). In addition, fasted female *Casp2*^*−/−*^ mice, also showed increased levels of fatty acid-binding protein 4 (FABP4), peroxisome proliferator-activated receptor-*α* (PPAR*α*), PPAR*γ* coactivator 1*α* (PGC1*α*), PGC1*β* and ACOX1 (Figure 3d). Combined with the lack of fasting-induced increase in serum glycerol levels, the data suggest that female *Casp2*^*−/−*^ mice have altered utilization of glyceroneogenesis for recycling of fatty acids and increased fatty acid oxidation in WAT. In contrast, in the fed state male *Casp2*^*−/−*^ mice had significantly reduced levels of the mitochondrial fatty acid transporter, carnitine/palmitoyl-CoA transferase (CPT1a) similar to those found in fasted WT mice (Figure 3c). This suggests that mitochondrial *β*-oxidation of FFAs may be decreased in gWAT of male *Casp2*^*−/−*^ mice.

Consistent with other known gene expression changes following lipolysis, levels of fatty acid synthase (FASN) and the glucose-transport 4 (Glut4) were decreased in all groups following fasting (Figures 3c and e). Gene expression of the adipokine leptin was decreased in male but not in female *Casp2*^*−/−*^ mice; however, no significant differences in circulating leptin levels were identified in the fed animals (Figures 3a and d; data not shown).

### Caspase-2-deficiency does not alter fasting-induced hepatosteatosis or metabolic gene expression

**Figure 4:**
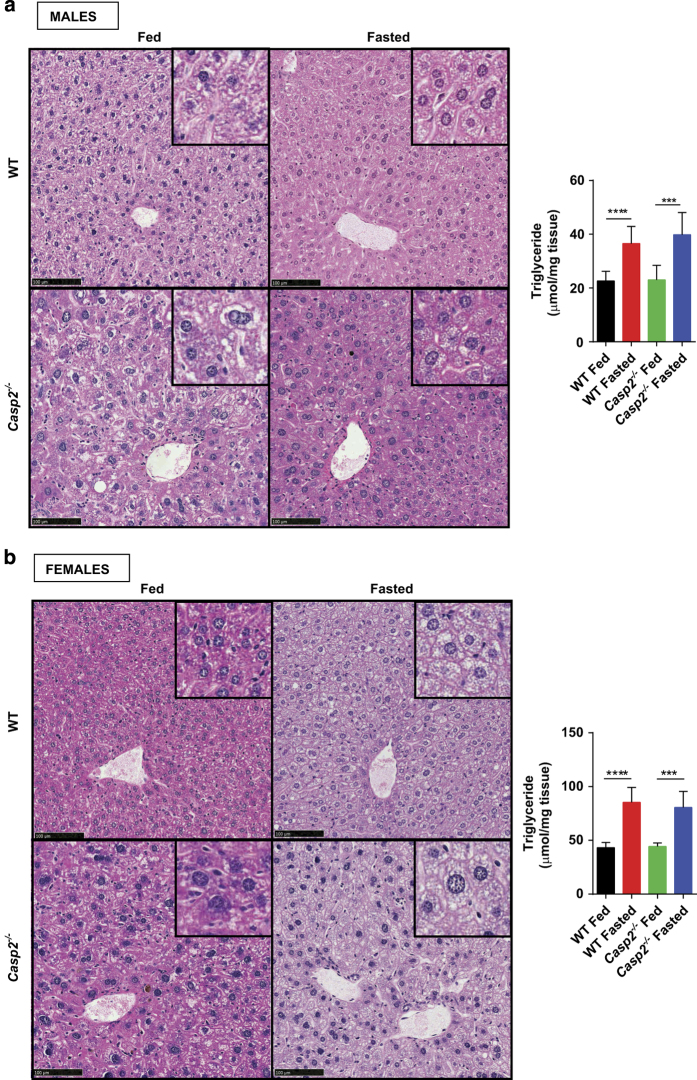
Fasting-induced hepatosteatosis develops in WT and *Casp2*^*−/−*^ mice. Histological analysis of liver from (**a**) male and (**b**) female fed and fasted WT and *Casp2*^*−/−*^ mice (×20 magnification). Inset boxes display zoomed in region of image. Bar graphs show liver triglyceride levels as measured by biochemical assay. Values are mean±S.D. (*n*=4–8). Values are mean±S.D. (*n*=4–8). Unpaired *t*-test: ***P<0.001 and ****P<0.0001. Scale bar, 100 *μ*m.

Prolonged fasting in mice is known to reduce hepatocyte cell size and liver mass.^26^ Although this may be accounted for by a decrease in protein liver content, we first wanted to determine other possible reasons for why fasting liver mass differed between male WT and *Casp2*^*−/−*^ mice. Histological analysis of liver, demonstrating lipid droplet accumulation in fasted hepatocytes, and measurement of liver triglycerides showed no difference between genotypes (Figures 4a and b). This indicates that *Casp2* does not affect fasting-induced hepatosteatosis in male and female mice. Glycogen content can also influence liver mass and was therefore determined in male mice; however, there was high variability with no difference between genotypes (data not shown). These results indicate that the difference in fasted male liver mass is not due to altered glycogen content or altered lipid uptake.

**Figure 5:**
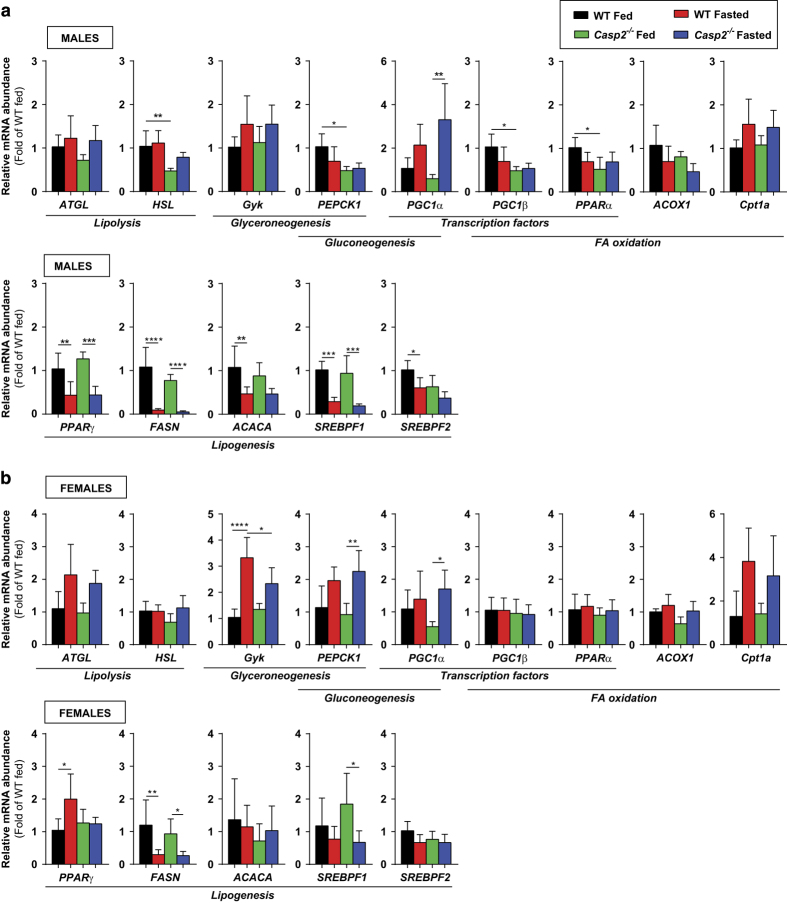
Metabolic gene expression in the liver of fed and fasted WT and *Casp2*^*−/−*^ mice. Metabolic gene expression in the liver of (**a**) male and (**b**) female fed and fasted mice. Values are mean±S.D. (*n*=4–8). One-way ANOVA: *P<0.05, **P<0.01, ***P<0.001 and ****P<0.0001.

Fasting stimulates the liver at the level of gene expression to increase pathways of gluconeogenesis while decreasing lipogenesis.^17^ Consistent with this, in male and female WT and *Casp2*^*−/−*^ mice, fasting decreased hepatic gene expression of lipogenic genes while increasing expression of gluconeogenic PGC1*α* (Figures 5a and b). In addition, PPAR*γ* was decreased in male WT and male and female *Casp2*^*−/−*^ mice but not WT females (Figures 5a and b). Differences between genotypes were minimal, particularly in females, but interestingly in fed *Casp2*^*−/−*^ males, hepatic levels of HSL, PPAR*α* and PGC1*β* were significantly decreased compared to WT mice (Figure 5a). However, as ketone body production is similar to WT mice (Figure 2c), it is unclear if liver FA oxidation is affected. In contrast to gWAT, levels of *Cpt1a* were not altered in the liver of male *Casp2*^*−/−*^ mice (Figure 5a).

### Caspase-2 regulates fasting-induced autophagy in the liver and skeletal muscle

**Figure 6:**
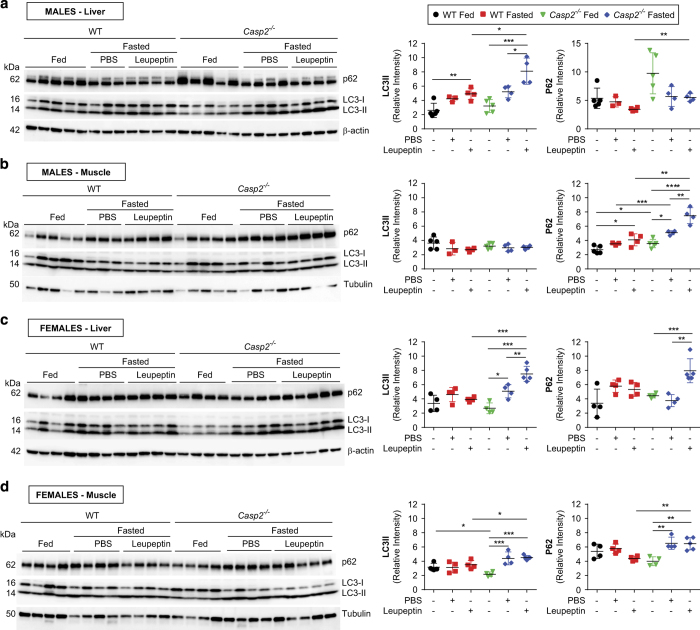
Caspase-2 alters fasting-induced autophagy in the liver and skeletal muscle. Immunoblot analysis of LC3 and p62 in the liver and muscle from (**a** and **b**) male and (**c** and **d**) female fed and fasted WT and *Casp2*^*−/−*^ mice treated with or without leupeptin. Scatter plots display densitometry analysis of LC3II and P62 as determined using stain-free gels as described in the Materials and Methods section. Tubulin and *β*-actin are provided as visual loading controls only. Values are mean±S.D. (*n*=4–5). One-way ANOVA (within genotype) or unpaired *t*-test (pair-wise comparison between genotype): *P<0.05, **P<0.01, ***P<0.001 and ****P<0.0001.

Fasting-induced autophagy has been shown to play an important role in the proteolytic breakdown of liver and skeletal muscle for energy supply and maintenance of blood glucose levels.^21,22^ Increased autophagy also contributes to a decrease in liver protein content that, along with inhibition of protein synthesis, can contribute to a decrease in liver mass during fasting.^26^ Therefore, to investigate if autophagy was contributing to the phenotype of male *Casp2*^*−/−*^ mice, we assessed autophagic flux by immunoblot analysis of autophagy proteins, LC3 and p62, after i.p. injection of leupeptin in fasted mice (Figure 6). Leupeptin suppresses autophagosome degradation by the lysosome thereby increasing the reliability of measuring LC3II (located on the autophagosomal membrane) protein as a marker of autophagy.^21^ Compared to WT mice, fasting-induced autophagy indicated by increased LC3II protein, was significantly higher in both male and female *Casp2*^*−/−*^ liver and female quadricep skeletal muscle (Figures 6a–c). In WT mice, fasting increased autophagy-associated LC3II levels in male livers only (Figure 6a). In *Casp2*^*−/−*^ females, expression of p62, an interactor of LC3 on autophagosomes that increases following prolonged starvation,^27^ followed a similar pattern to that of LC3II (Figures 6c and d). In male skeletal muscle, p62 levels were significantly higher in *Casp2*^*−/−*^ mice compared to WT mice, even in the fed state. Interestingly, p62 levels were significantly increased by fasting in male WT and *Casp2*^*−/−*^ skeletal muscle, despite no differences in LC3II being detected between all groups (Figure 6b).

### AMPK and mTOR pathways in fed and fasted liver and skeletal muscle

**Figure 7:**
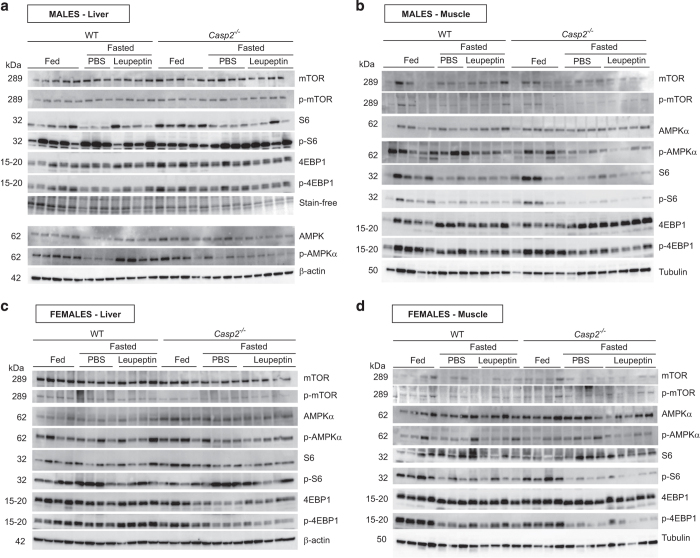
Analysis of AMPK and mTOR pathways in fed and fasted liver and skeletal muscle. Immunoblot analysis of total and phosphorylated forms of mTOR, AMPK*α*, S6 and 4EBP1 in the liver and muscle from (**a** and **b**) male and (**c** and **d**) female fed and fasted WT and *Casp2*^*−/−*^ mice treated with or without leupeptin. Tubulin and *β*-actin are provided as visual loading controls only.

Autophagy is regulated by a number of signaling pathways that respond to fasting, including the AMP-activated protein kinase (AMPK; activator of autophagy) and mechanistic target of rapamycin (mTOR; suppressor of autophagy) pathways. In general, fasting results in activation of AMPK which, via negative feedback, inhibits mTOR and thus autophagy. A previous report suggested that autophagy enhanced in the absence of *Casp2* involves AMPK and mTOR signaling.^24^ We therefore assessed the activation of these pathways in our samples to see whether they contributed to the observed differences in levels of autophagy (Figure 7). As demonstrated by immunoblot analysis, loss of *Casp2* in both male and female mice did not alter the activation of mTOR or AMPK*α* in muscle or liver tissue (Figure 7 and Supplementary Figure S3). However, in our hands phosphorylated and total mTOR were poorly detected in all quadricep skeletal muscle samples. It should be noted that in fasted male WT liver, p-AMPK*α* levels were clearly increased following leupeptin injection (Figure 7a and Supplementary Figure S3a). Phosphorylation of downstream targets of mTOR, including S6 ribosomal protein and eukaryotic translation initiation factor (4EBP1), were also assessed. There were no substantial differences in S6 activation (p-S6/S6) but fasting decreased activation of 4EBP1 (p-4EBP1/4EBP1) in male WT liver, and male and female skeletal muscle (Figure 7 and Supplementary Figure S3). Furthermore, in male and female leupeptin-treated skeletal muscle, loss of *Casp2* resulted in a more substantial decrease in p-4EBP1 following fasting when compared with fasted WT mice (Supplementary Figures S3b and d). This suggests, at least in this tissue, that inhibition of downstream components of the mTOR pathway may contribute to increased autophagy in the absence of *Casp2*. Interestingly, fasting appeared to increase p-S6 in both male and female livers (Figures 7a and d). Paradoxical increases in p-S6 following fasting have been previously observed in the liver.^26^

## Discussion

Body composition is known to influence glucose tolerance and homeostasis with ageing. In general, females are known to have higher body fat and altered fat distribution as compared to males but are more insulin sensitive and have lower fasting blood glucose levels and better glucose tolerance with age.^28,29^ In the present study, we show that the previously observed improved glucose homeostasis of male *Casp2*^*−/−*^ mice^10^ is likely independent of insulin sensitivity and does not occur in female *Casp2*^*−/−*^ mice. We have further demonstrated sex-specific differences in body composition and lipid metabolism with male mice having increased lipolysis of adipose tissue upon fasting whereas female *Casp2*^*−/−*^ mice have reduced adipocyte mass in both the fed and fasted states. For the first time, we also demonstrate an *in vivo* role for *Casp2* in the response to fasting-induced autophagy in the liver and skeletal muscle.

Sex-specific differences in glucose tolerance are known to occur in other strains of mice. For example, male haplo-insufficient IGF-1 receptor (*Igf1r*^*+/*^*^−^*) mice (on the same C57BL/6 background as *Casp2*^*−/−*^ mice), become glucose intolerant with age while female *Igf1r*^*+/*^*^−^* do not.^30^ In addition, male, but not female, haplo-insufficient *Nedd4*^*+/*^*^−^* mice have lower fasting blood glucose levels.^31^

As body composition differences were observed in both male and female mice, it was thought unlikely that body composition in male mice was the predominant reason for improved glucose homeostasis with age. Differences in autophagic flux were also considered a possible reason for improved glucose tolerance in male *Casp2*^*−/−*^ mice. However, autophagy did not differ from WT mice in the fed state and fasting increased autophagy independent of gender in *Casp2*^*−/−*^ mice. Regardless, the level of autophagy in male *Casp2*^*−/−*^ mice was still significantly higher than their WT counterparts. In the absence of differences in fasting hepatic triglycerides or glycogen content, this suggests that the decreased fasting liver mass is due to increased autophagic proteolysis of liver tissue.

Increased uptake of glucose into skeletal muscle was also considered; however, this is unlikely due to our previous observation of decreased epidermal skeletal muscle in aged males only.^9^ In addition, we observed no difference in *Glut4* expression in the quadriceps of *Casp2*^*−/−*^ mice; however, this is not an accurate representation of Glut4 function in skeletal muscle, which often differs in cellular distribution and transport rather than at expression level. Future studies will be needed using precise approaches to clearly determine the contribution of glucose uptake and utilization by both the liver and skeletal muscle.

The increase in fasting-induced lipolysis of adipose tissue in male *Casp2*^*−/−*^ mice, and the differing metabolic gene expression profiles between male and female *Casp2*^*−/−*^ mice suggest that variance in fuel utilization may be the primary reason for altered glucose homeostasis in males. Fasting is known to result in higher serum glycerol levels in females compared to males following fasting.^32^ However, in female *Casp2*^*−/−*^ mice, glycerol did not increase following fasting, despite similar level of increased FFAs and ketone bodies compared to WT mice. Gene expression analysis of WAT suggests that glyceroneogenesis, a prominent pathway involved in balancing the release of FFAs and glycerol from WAT during fasting,^19^ may be enhanced in female *Casp2*^*−/−*^ mice such that the release of glycerol into circulation is lower due to it being re-esterified back to triglycerides. Another possibility for lower fasting serum glycerol is that hepatic glycerol uptake is increased in fasted female *Casp2*^*−/−*^ mice; however, this was not investigated in this study. In addition, we detected no difference in gene expression of glyceroneogenic or gluconeogenic enzymes, which may indicate an increased utilization of glycerol in the liver.

Tiwari *et al*.^23^ previously reported that the steady state level of basal autophagy was increased by loss of *Casp2* in isolated primary MEFs, astrocytes, neurons, osteoclasts and cerebral cortex tissue. Here, by using leupeptin as means of stabilizing LC3II levels, we have shown that *in vivo* loss of *Casp2* significantly enhances fasting-induced autophagy in the liver and skeletal muscle. While basal levels of autophagy appeared to be unaltered in the fed state of *Casp2*^*−/−*^ mice this group was not treated with leupeptin, which may have allowed for subtle differences to be detected. Tiwari *et al*. reported that loss of *Casp2* modulation of autophagy involves both the AMPK and mTOR pathways; however, we found no conclusive evidence to support this in the liver and skeletal muscle.

In conclusion, this study shows that *Casp2* influences glucose homeostasis and lipid metabolism in a sex-specific manner and is involved in the regulation of fasting-induced autophagy to meet energy demands.

## Materials and Methods

### Animals

Male and female WT and *Casp2*^*−/−*^ mice on a C57BL/6J background^9,33^ were used for experimental studies at 18–22 months of age. Ethics for approval for research using animals was obtained from SA Pathology/Central Northern Adelaide Health Services Animal Ethics Committee, in accordance with National Health and Medical Research Council of Australia guidelines. Mice were housed in pathogen-free conditions with a 12-h light-dark cycle (lights on at 0600 hours) with *ad libitum* access to water and standard chow. Food intake was monitored over a 5-day period. Mice were killed at the same time of day in the light phase (1100 to 1300 hours) in fed (*ad libitum*) or fasted (18 h) state. Mice were provided *ad libitum* access to drinking water. For assessment of autophagic flux, 18-h fasted mice received an i.p. injection of a leupeptin solution at a dose of 15 mg/kg body weight or PBS as control, 1 h prior to killing the mice. All animals were anesthetized, blood was collected by cardiac puncture and killed by cervical dislocation. Collected tissues and serum were snap-frozen in liquid nitrogen and maintained at −80 °C until analyzed.

### Liver and serum biochemistry

Triglycerides were determined in the serum by automated analysis (SA Pathology, Adelaide, SA, Australia). FFAs (serum), glycerol (serum), triglycerides (liver) and glycogen (liver) were determined using commercially available assay kits (BioVision, Milpitas, CA, USA; Sigma, St. Louis, MO, USA).

### Intraperitoneal glucose tolerance test (IPGTT) and insulin tolerance test (IPITT)

Glucose and insulin tolerance tests were performed as previously described,^10^ by injecting glucose (i.p.) at 1 mg/kg body weight or insulin (i.p.) at 0.5 U insulin/kg body weight.

### Real-time quantitative PCR (qPCR)

Total RNA was extracted from frozen tissue, reverse transcribed and real-time qPCR performed as previously described.^10^ Gene expression was normalized to housekeeping gene (*β*-actin or TATA-binging protein (TBP)) and then expressed as fold change of WT mice fed using the 2^−∆∆Ct^ method. See Supplementary Table S1 for primer sequences.

### Immunoblotting and stain-free densitometry

Total proteins were isolated from tissues using RIPA buffer with the addition of Halt protease and phosphatase inhibitor cocktail (Thermo Fisher, Rockford, IL, USA) as previously described.^10^ Proteins (35 *μ*g) were resolved by 4–20% Criterion TGX Stain-Free Precast Midi Protein Gel (Bio-Rad, Hercules, CA, USA), transferred to PVDF membrane and then imaged using ChemiDoc Touch Imaging System (Bio-Rad) to allow for stain-free normalization and quantification of proteins of interest.^34,35^ Specific proteins were detected using the following primary antibodies: AMPK*α* (2793), p-AMPK*α* (Thr172, 40H9 #2535), 4EBP1 (53H11 #9644), phospho-4BEP1 (Thr37/46, 236B4 #2855), mTOR (#2983), phospho-mTOR (ser2481 #2974), S6 ribosomal protein (5G10 #2217), phospho-S6 ribosomal protein (Ser240/244, D68F8) and XP (#5364; Cell Signaling Technology, Beverly, MA, USA); *β*-actin and clone AC-15 (#A5441; Sigma-Aldrich, St Louis, MO, USA); *α*-tubulin (#ab18251; Abcam, Cambridge, MA, USA); caspase-2 (clone 11B4); LC3B/MAP1LC3B (NB100-2220; Novus Biologicals, Littleton, CO, USA); and p62/SQSTM1 (M01) clone 2C11 (#H00008878-M01; Abnova, Littleton, CO, USA).

### Histological analysis

Tissues fixed in HistoChoice (Sigma-Aldrich), were embedded in paraffin, sectioned and stained with hematoxylin and eosin. Adipocyte areas in gWAT were manually traced and quantified using ImageJ software (NIH, Bethesda, MD, USA).

### Statistical analyses

Statistical analysis was performed using GraphPad Prism software (v 6.0, San Diego, CA, USA). Data are expressed as mean±S.D. or mean±S.E.M. For pair-wise comparisons, a two-tailed unpaired *t*-test with Welch’s correction was used. For multi-group comparisons, one-way ANOVA was used with Tukey’s *post hoc* testing unless stated otherwise.

## Abbreviations

4EBP1: eukaryotic translation initiation factor 4E-binding protein
AMPK: AMP-activated protein kinase
BAT: brown adipose tissue
*β*-HB: *β*-hydroxybutyrate
Casp2: caspase-2
FASN: fatty acid synthase
FFAs: free fatty acids
gWAT: gonadal white adipose tissue
iBAT: interscapular brown adipose tissue
IPGTT: intraperitoneal glucose tolerance test
IPITT: intraperitoneal insulin tolerance test
LC3: microtubule-associated protein 1A/1B light chain 3
mTOR: mechanistic target of rapamycin
WAT: white adipose tissue.
