# Supplementary material for: Sex-specific alterations in glucose homeostasis and metabolic parameters during ageing of caspase-2-deficient mice
Source: Cell Death Discov. 2016 Feb 29;2:16009–. doi: 10.1038/cddiscovery.2016.9 (PMC4979492; doi:10.1038/cddiscovery.2016.9)
Supplement: Supplementary Information [file cddiscovery20169-s1.pdf]

SUPPLEMENTARY INFORMATION

Sex-specific alterations in glucose homeostasis and metabolic parameters during ageing of Caspase-2-deficient mice

Claire H Wilson[1]*, Andrej Nikolic[1], Stephen J Kentish[2,3], Sonia Shalini[1], George Hatzinikolas[2], Amanda J Page[2,3], Loretta Dorstyn[1], Sharad Kumar[1]*

[1] Centre for Cancer Biology, University of South Australia, Adelaide, SA 5001, Australia

[2] Discipline of Medicine, University of Adelaide, Adelaide, SA 5000, Australia

[3] South Australian Health and Medical Research Institute, Adelaide, SA 5000, Australia

SUPPLEMENTARY FIGURE LEGENDS

Supplementary Figure S1. Food intake is not altered in aged *Casp2*$^{-/-}$ mice. Average daily food intake of (a) male and (b) female fed WT and *Casp2*$^{-/-}$ mice. Values are means ± S.D. (n=8-9)

Supplementary Figure S2. Decreased lipid droplet size in iBAT of fasted male *Casp2*$^{-/-}$ mice. Histological analyses of iBAT from (a) male and (b) female fed and fasted WT and *Casp2*$^{-/-}$ mice (20x magnification). Inset boxes display zoomed in region of image. Images are representatives from individual mice. (Scale bars: 100 µM).

Supplementary Figure S3. Densitometry analysis of AMPK and mTOR immunoblots. Densitometry of immunoblots displayed in figure 6. Phosphorylated/total forms of MTOR in liver of (a) male and (c) female mice and phosphorylated/total forms of AMPKα, S6 and 4EBP1 in liver and muscle from (a, b) male and (c,d) female fed and fasted WT and *Casp2*$^{-/-}$ mice treated with or without leupeptin. Densitometry determined using stain-free gels as described in materials and methods. Values are means ± S.D. (n=4-5). One-way ANOVA (within genotype) or unpaired *t*-test (pair-wise comparison between genotype): $p < 0.05$, $**p < 0.01$, $***\ p < 0.001$.

Supplementary Table S1. Sequences of primers used for qPCR analysis

**Gene**	**Forward primer (5'-3')**	**Reverse primer (5' -3')**
ACACA	CGGGTGAAGTACATCAAGCG	ACTTGGTGTAGCTTCTCCCC
ACOX1	GCTGAGGAACCTGTGTCTCT	TCAAAGGCATCCACCAAAGC
ADIPONECTIN	TGACGACACCAAAAGGGCTC	CACAAGTTCCCTTGGGTGGA
ATGL	AACACCAGCATCCAGTTCAA	GGTTCAGTAGGCCATTCCTC
β-ACTIN	GATCATTGCTCCTCCTGAGC	AGTCCGCCTAGAAGCACTTG
CPT1A	CCTGGGCATGATTGCAAAG	GGACGCCACTCACGATGTT
FABP4	AAGGTGAAGAGCATCATAACCCT	TCACGCCTTTCATAACACATTCC
FASN	AGCTTCGGCTGCTGTTGGAAGT	TCGGATGCCTCTGAACCACTCACA
GLUT4	GTCCTCCTGCTTGGCTTCTT	AGCTGAGATCTGGTCAAACG
GYK	TGAACCTGAGGATTTGTCAGC	CCATGTGGAGTAACGGATTTCG
HPRT	GAGAGCGTTGGGCTTACCTC	CTAATCACGACGCTGGGACT
HSL	TGGTTCAACTGGAGAGCGGAT	TGATGCAGAGATTCCCACCTG
LEPTIN	CAGGATCAATGACATTTCACACA	GCTGGTGAGGACCTGTTGAT
PDK4	AGAAGACCAGAAAGCCCTGTCA	GCCATTGTAGGGACCACATTATG
PEPCK1	ATCATCTTTGGTGGCCGTAG	CATGGCTGCTCCTACAAACA
PGC1α	CGCCGTGTGATTTACGTTGG	GCTGTCTCCATCATCCCGC
PGC1β	TCTGACGTGGACGAGCTTTC	GTGCCATCCACCTTGACACA
PPARα	ATGCCAGTACTGCCGTTTTC	CCGAATCTTTCAGGTCGTGT
PPARγ	GTCACACTCTGACAGGAGCC	AGAACGTGACTTCTCAGCCC
SREBPF1	CTTTTCCTTAACGTGGGCCT	GCTGGAGCATGTCTTCGATGT
SREBPF2	CCCTGGAAGTGACCGAGAGT	GAGACTGCTCCACAGGTGAC
TBP	CAAACCCAGAATTGTTCTCCTT	ATGTGGTCTTCCTGAATCCCT

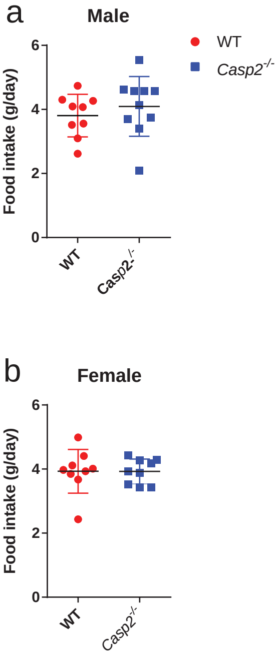

Supplementary Figure S1

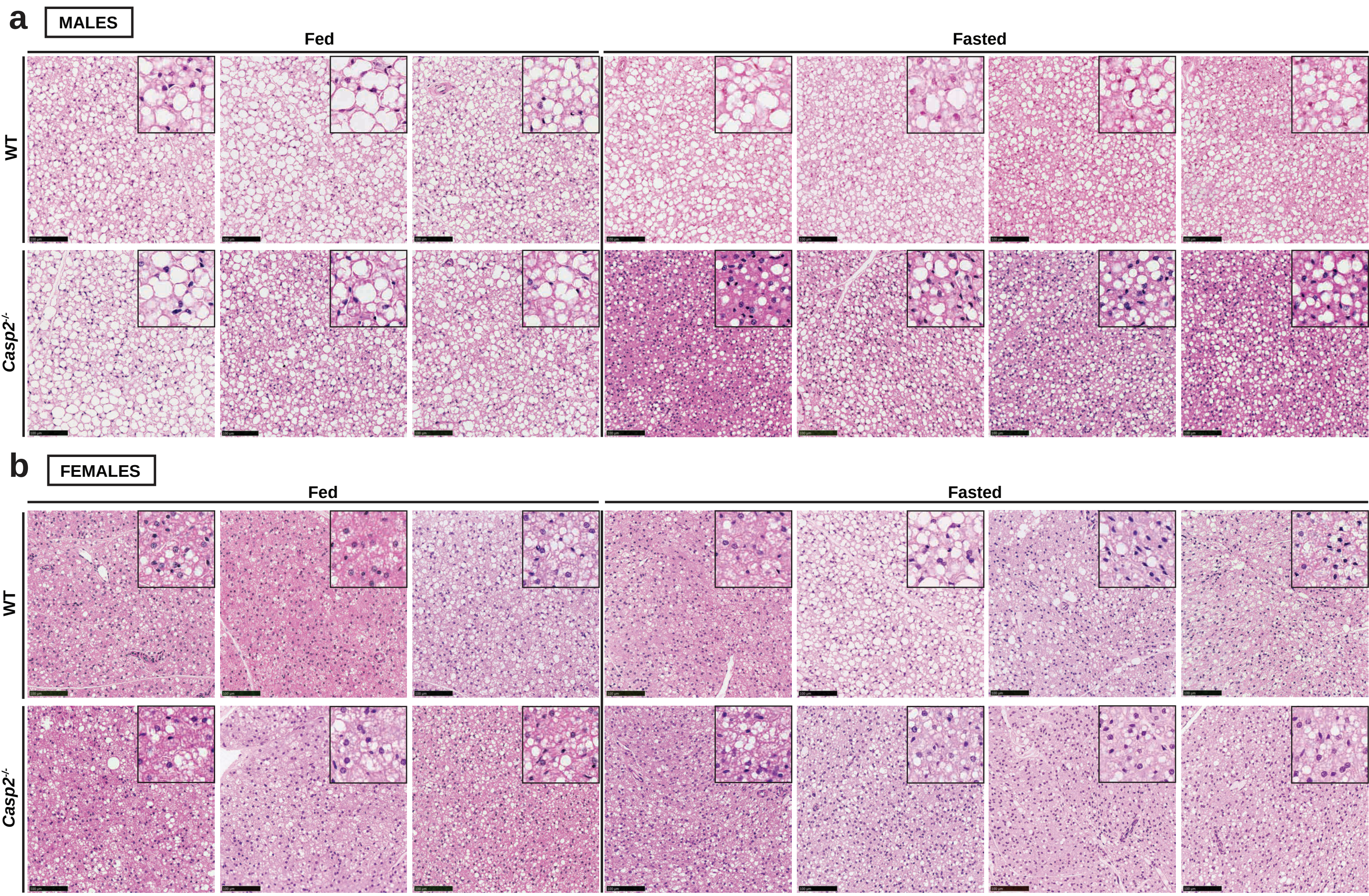

Supplementary Figure S2

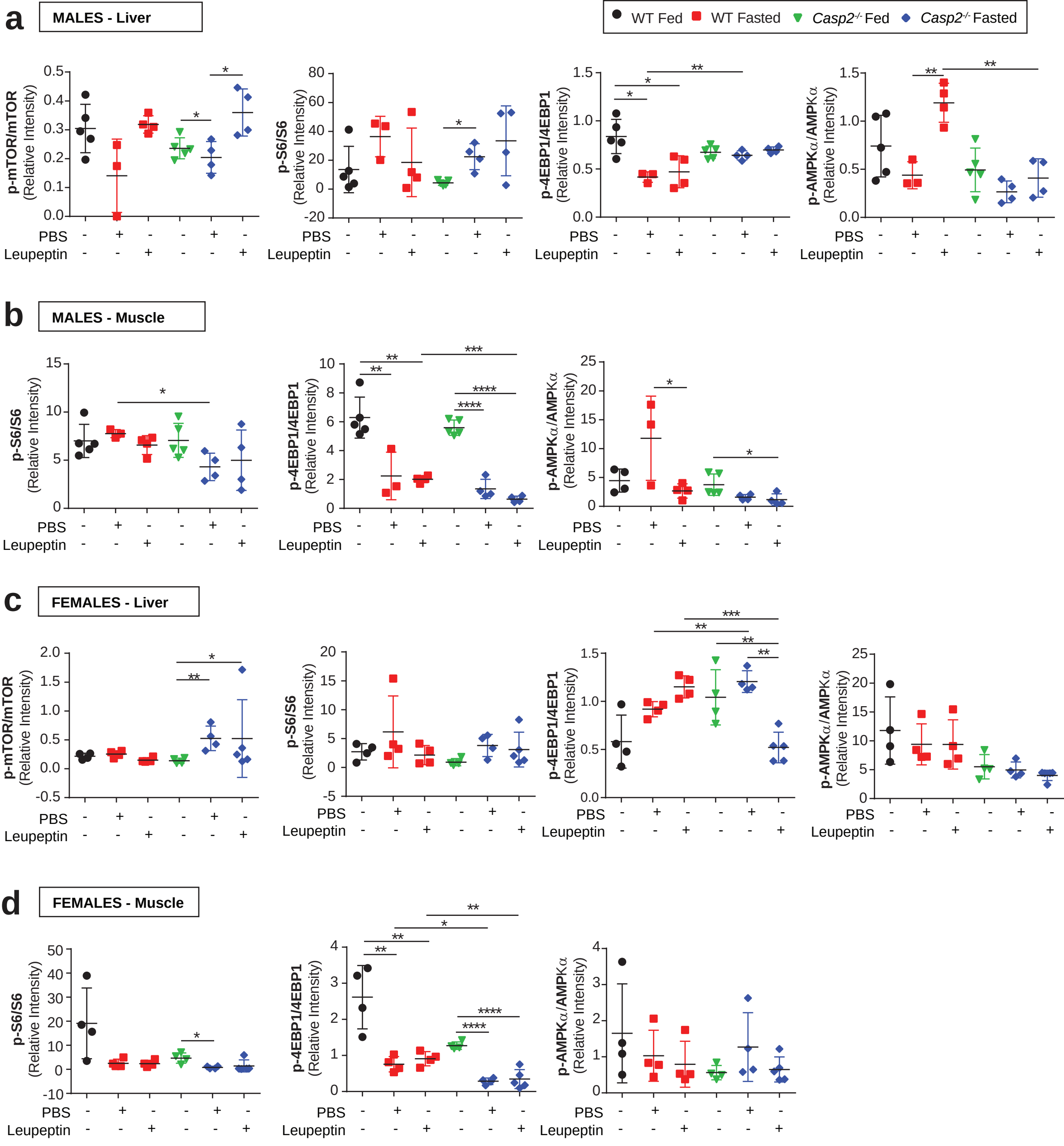

Supplementary Figure S3
