# Supplementary figures and images for: Sex-specific alterations in glucose homeostasis and metabolic parameters during ageing of caspase-2-deficient mice
Source: Cell Death Discov. 2016 Feb 29;2:16009–. doi: 10.1038/cddiscovery.2016.9 (PMC4979492; doi:10.1038/cddiscovery.2016.9)

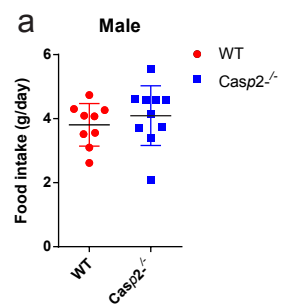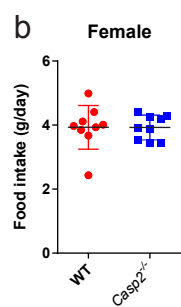

**Supplementary Figure S1**

Supplement: Supplementary Figure S1 [file cddiscovery20169-s2.pdf]

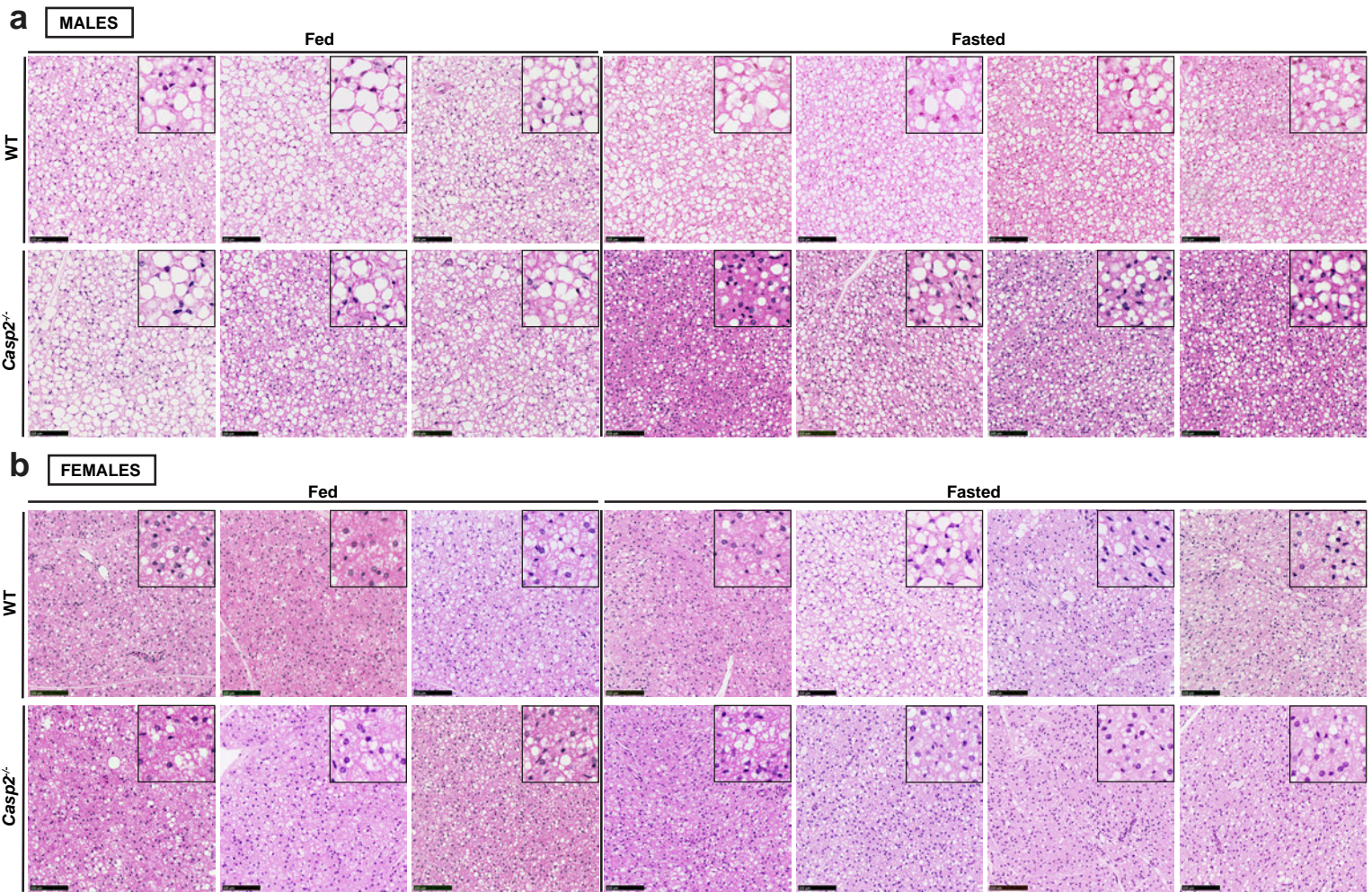

**Supplementary Figure S1**

Supplement: Supplementary Figure S2 [file cddiscovery20169-s3.pdf]

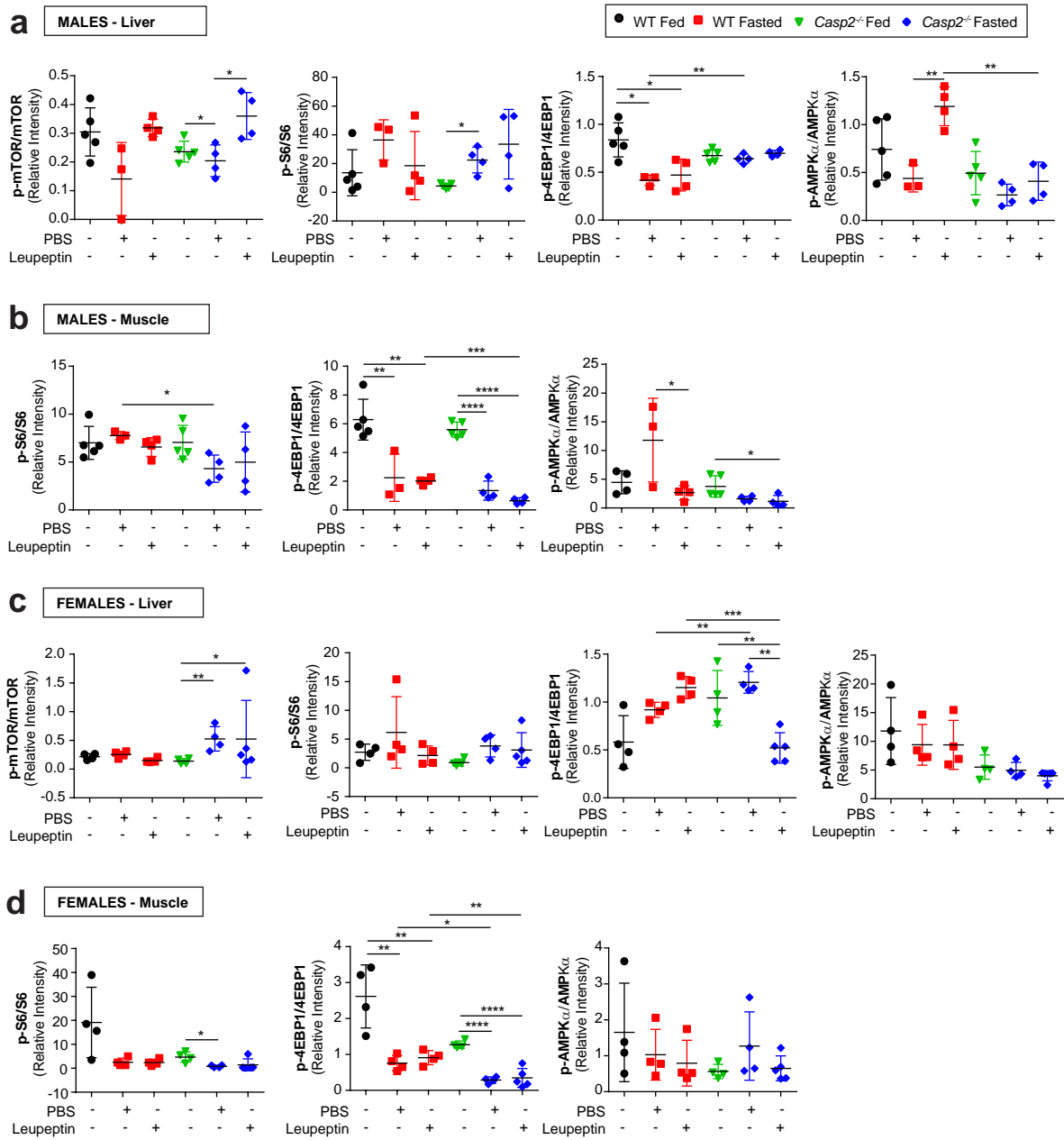

Supplementary Figure S1

Supplement: Supplementary Figure S3 [file cddiscovery20169-s4.pdf]
